# Efficient Optomechanical Mode-Shape Mapping of Micromechanical Devices

**DOI:** 10.3390/mi12080880

**Published:** 2021-07-27

**Authors:** David Hoch, Kevin-Jeremy Haas, Leopold Moller, Timo Sommer, Pedro Soubelet, Jonathan J. Finley, Menno Poot

**Affiliations:** 1 Department of Physics, Technical University of Munich, 85748 Garching, Germany; david.hoch@tum.de (D.H.); ge56kih@mytum.de (K.-J.H.); ga48tig@mytum.de (L.M.); timo.sommer@tum.de (T.S.); Pedro.Soubelet@wsi.tum.de (P.S.); finley@wsi.tum.de (J.J.F.); 2 Munich Center for Quantum Science and Technology (MCQST), 80799 Munich, Germany; 3 Institute for Advanced Study, Technical University of Munich, 85748 Garching, Germany; 4 Walter Schottky Institute, Technical University of Munich, 85748 Garching, Germany

**Keywords:** optomechanics, mode-mapping, MEMS, phase-lock loop, silicon-nitride, membrane

## Abstract

Visualizing eigenmodes is crucial in understanding the behavior of state-of-the-art micromechanical devices. We demonstrate a method to optically map multiple modes of mechanical structures simultaneously. The fast and robust method, based on a modified phase-lock loop, is demonstrated on a silicon nitride membrane and shown to outperform three alternative approaches. Line traces and two-dimensional maps of different modes are acquired. The high quality data enables us to determine the weights of individual contributions in superpositions of degenerate modes.

## 1. Introduction

In recent years, there have been many applications for integrated opto- and electromechanics extending from, e.g., mobile communication [1] and highly sensitive sensors [2,3,4,5,6,7,8] to position detection close to the quantum limit [9,10,11]. In the development of such devices, an efficient method for mode characterization is instrumental and, hence, a number of techniques including optical interferometry [12,13,14], heterodyne detection [15,16], dark field imaging [8,12], and force microscopy [1,10,17,18] have been developed to visualize mechanical modes. However, most of these have one or more drawbacks, such as poor sensitivity, lacking phase information, low spatial resolution, or long measurement times. Here, we demonstrate an experimental method that combines the high sensitivity of the optical interferometric techniques with demodulation and frequency tracking to offer rapid and robust imaging of multiple modes simultaneously. The advantages of the technique, combining high resolution and sensitivity, as well as its robustness against sign changes of the mode shape at the nodal lines while tracking the resonance frequency via a phase-lock loop (PLL), are illustrated by mapping the eigenmodes of a square silicon nitride (SiN) membrane. With our method, the eigenmodes of the membrane can not only be unambiguously identified but also their mode composition can be determined quantitatively and insights in clamping losses are provided.

## 2. Materials and Methods

**Figure 1:**
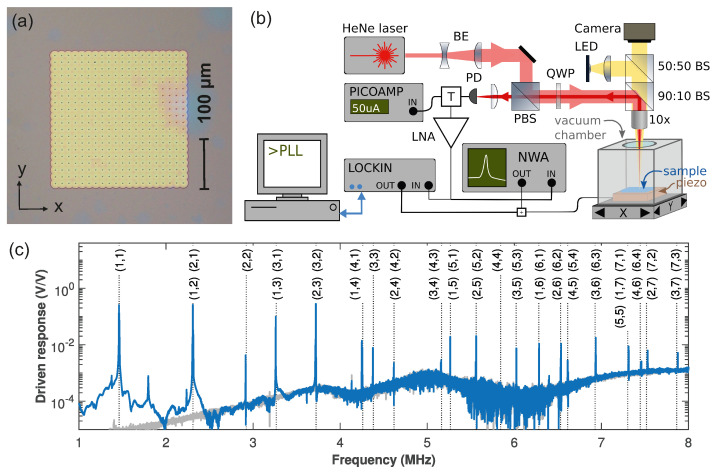
(**a**) Optical micrograph of the membrane (**b**) Schematic overview of the measurement setup. BE: beam expander, (P)BS: (polarizing) beam splitter, QWP: quarter wave plate, PD: photodetector, LNA: low-noise amplifier, NWA: network analyzer, T: bias tee, +: combiner, LED: light emitting diode for illumination. (**c**) Driven response of the membrane measured using the NWA (blue) with the calculated frequencies (Equation (2)) as black dashed lines and the mode numbers indicated. The gray trace is the instrument background.

Before discussing the results of our method in Section 3 in depth, first the sample and setup are briefly discussed. The SiN membranes are made on chips with 330 nm high-stress Si_3_N_4_ [19,20,21]. As detailed in Appendix C, release holes are defined using electron-beam lithography followed by a fluorine-based reactive ion etch, exposing the underlying SiO_2_ [22]. The membranes are released using buffered hydrofluoric acid followed by critical-point drying; a micrograph of the final suspended membrane is shown in Figure 1a. The reflectivity of the structure depends on the distance between the membrane and Si substrate, enabling interferometric measurements of the membrane displacement using the setup shown in Figure 1b. For this, a HeNe laser is focused using a 10× microscope objective with a 32 mm working distance and NA = 0.28. It has a fixed position outside the vacuum chamber, which is mounted on a motorized x-y stage that can scan with steps of 1.25 μm while measuring the reflected light using a photodetector. For excitation and detection, either a network analyzer (NWA, HP 4396A) or lock-in amplifier (LIA, Zurich Instruments HF2) can be used. Their output goes to the piezo-electric actuator to excite the membrane. Its vibrations modulate the light on the photodetector, which is again detected with the NWA or LIA. The dc reflection can be recorded using a picoamp current meter. For further details on the analysis of the signal, see Appendix A.

## 3. Results

The out-of-plane modes of a square membrane with side lengths *a* under uniform tension can be calculated analytically [23]. The normalized mode shapes are:

(1) $$\xi_{m,n}\left(x,y\right)=\sin\left(\pi mx/a\right)\sin\left(\pi ny/a\right),$$

so that the local displacement is [24] $u_{m,n}\left(x,y\right)=U_{m,n}\xi_{m,n}\left(x,y\right)$. The modes are labeled using two integers *m* and *n* that count the number of anti-nodes in the x and y direction, respectively. The (m,n) mode, thus, has m−1 (n−1) vertical (horizontal) nodal lines. At the anti-nodes, $\xi_{m,n}=1$ and the amplitude is $U_{m,n}$. The corresponding eigenfreqencies are:

(2) $$f_{m,n}=f_{1,1}\times {\left(\frac{m^{2}+n^{2}}{2}\right)}^{1/2};\;\;f_{1,1}=\frac{1}{2a}{\left(\frac{2\sigma }{\rho }\right)}^{1/2}.$$

Here, $\rho =3.17\times 10^{3}\;\;\mathrm{kg}/m^{3}$ is the mass density of Si_3_N_4_ [25] and σ∼1.05 GPa is the film stress in our wafers [20], yielding $f_{1,1}=1.48\;\;\mathrm{MHz}$ for a=275 μm.

**Figure A1:**
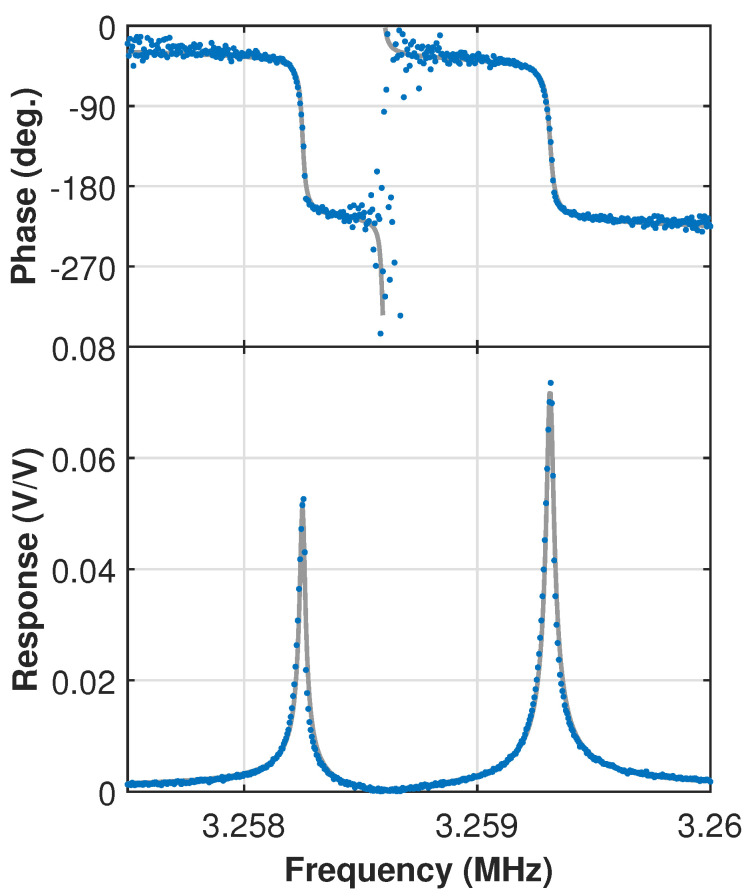
Network analyzer measurement of the driven response near the (3,1) and (1,3) eigenfrequencies. Two separate resonances with a spacing of 1.06 kHz can be seen. As discussed in the text, the left and right resonance corresponds to the (3,1) and (1,3) mode, respectively.

Experimentally, the eigenfrequencies appear as a series of sharp resonances in Figure 1c. The first peak is at 1.46 MHz, close to the result from Equation (2), which also shows that once $f_{1,1}$ is known, the other eigenfrequencies can be calculated; their values (dashed lines) nicely match the observed peaks so that resonances can be identified. For example, the peak at 2.92 MHz matches $f_{2,2}$. On the other hand, the one at 3.26 MHz coincides with both (1,3) and (3,1). Theoretically, a perfectly square membrane has degenerate modes, i.e., $f_{m,n}=f_{n,m}$ but, in practice, small imperfections can break the degeneracy. When zooming in, two peaks with ∼1 kHz splitting are visible (Figure A1). Still, from their frequencies alone these cannot be identified. Instead, their mode shape should be measured to unambiguously determine which peak corresponds to which mode.

### 3.1. Comparison of Different Methods

**Figure 2:**
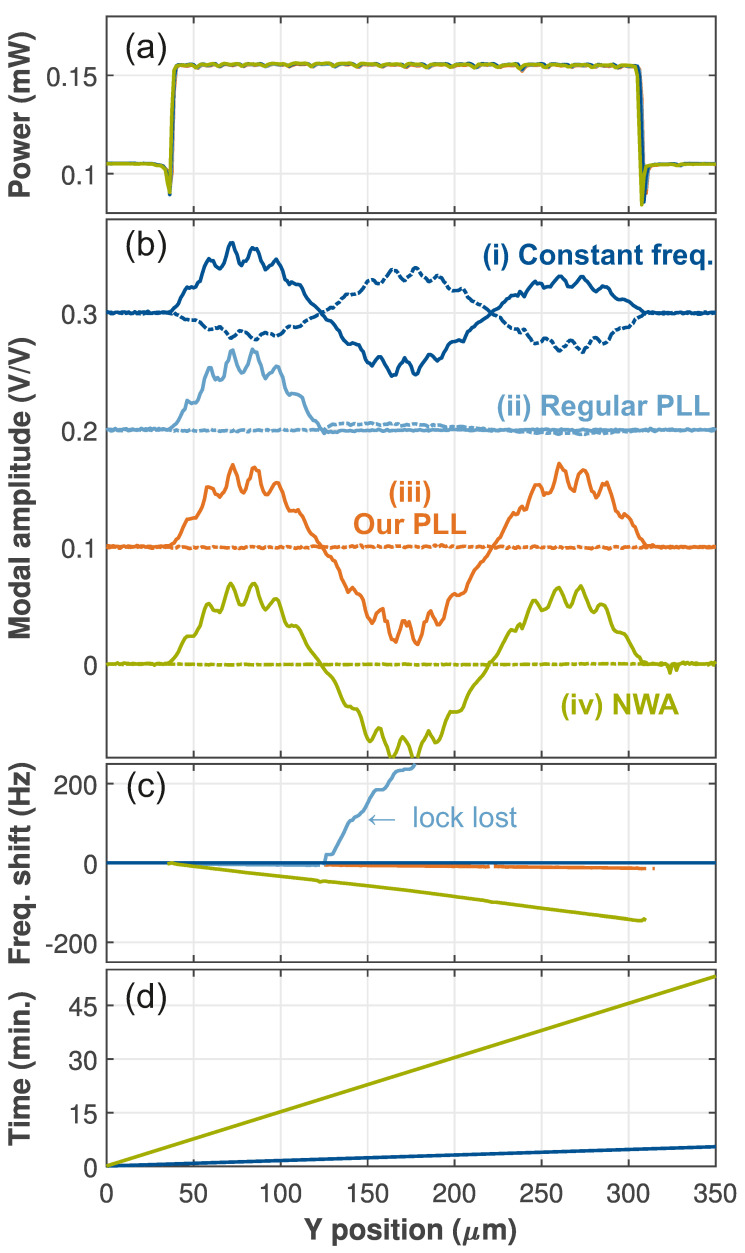
Line traces over the membrane for (i) constant frequency (dark blue), (ii) regular PLL (light blue), (iii) the modified PLL with mod 180° (orange), (iv) data measured using a network analyzer (green). (**a**) Reflected laser power. (**b**) The mode profile of the (1,3) mode near 3.26 MHz. Solid (dashed) lines indicate the real (imaginary) part. The curves are offset for clarity. (**c**) Frequency change during the measurement. For (i)–(iii), this was the actuation *f*, whereas for (iv) the resonance $f_{0}$ was extracted from the NWA traces. By definition the frequency was constant in (i). (**d**) Elapsed time since the start of the trace.

Before presenting the full two-dimensional mode maps, we first compare in Figure 2 line traces taken with different methods to show the robustness and efficiency of ours. The membrane is scanned in the *y* direction while sequentially acquiring the signal of the 3.260MHz mode using four different methods. First of all, Figure 2a shows the dc reflection, which overlaps for all methods. The suspended membrane has a higher reflectivity compared to the supported regions and the holes are visible as small dips in the signal.

Now, method (i) for obtaining a mode shape (dark blue lines in Figure 2) is to simply drive the mechanical resonator at that resonance frequency and record the amplitude [26]. Figure 2b shows that the suspended part of the membrane has a clear response with small modulations due to the holes. There are two nodes in the modal amplitude vs. *y*, indicating that this is the (1,3) and not the (3,1) mode. Taking a closer look shows that, unlike for the theoretical prediction of Equation (1), the anti-nodes have unequal magnitudes. In addition, the modal amplitude shows an imaginary part (see Appendix A for details) that grows with time. This indicates that the resonance frequency drifted from the (fixed) driving frequency during the measurement. This means that the naive approach (i) does not yield accurate mode shapes.

A standard approach to track a resonance is a phase-lock loop (PLL) [6,27]. Here, it is a software-implemented PI-controller in LabVIEW (note that it is also possible to perform this task using the digital signal processor in the lock-in amplifier [28,29], which can further improve the operation speed) in combination with digital demodulation in the LIA. The PI-controller updates the driving frequency *f* to keep the phase ϕ at the setpoint $\phi_{\mathrm{sp}}$ using:

(3) $$f_{n+1}=f_{1}+Pe_{n}+I\sum_{j=1}^{n}e_{j}.$$

Here, $e_{n}=\phi_{n}-\phi_{\mathrm{sp}}$ is the error in the *n*-th sample, and *P* and *I* are the proportional and integral gain, respectively. Figure 2b shows that with this regular PLL (method (ii), light blue), the first anti node has a lower imaginary part compared to the previous method. However, as also indicated by the sudden large shift in Figure 2c, the PLL loses lock after the node where the mode changes sign, resulting in a π jump in ϕ. This problem that almost all off-the-shelf PLL-based systems have, motivates our improvements to the regular PLL. For our method (iii), we, first of all, added a modulo operation: $e_{n}\to e_{n}\;\mathrm{mod}\;\pi$. This way, the PLL can handle the sign flips and will remain locked irrespective if the motion is in phase or in anti-phase. A second addition is to turn the PLL off until a minimum signal magnitude is reached. This maintains the frequency while scanning, e.g., over the nodes. The orange curve in Figure 2b shows the result of our new method: The anti nodes are now equal in magnitude and the imaginary part stays very small. Our robust method (iii) thus faithfully maps the mode, even in the presence of frequency drifts, nodes, and sign changes.

The fourth mode-mapping approach, method (iv), is performed with the NWA [14]. Here, a full frequency response is measured at every point of the line trace, and its fitted maximum and phase (Appendix A) are used to reconstruct the modal amplitude. Similar to our improved PLL (cf. method (iii)), method (iv) is also capable of mapping a drifting mode accurately (Figure 2b,c, green). However, as Figure 2d shows, the NWA method is about ten times slower compared to all other methods. Although it can be considered the gold standard, method (iv) is too slow to do, e.g., full 2D mode maps efficiently. After comparing the results from the line traces taken under realistic conditions with the different methods, it is clear that our method (iii) is the preferred technique. To further demonstrate its use, we will now turn our attention to two-dimensional maps of the modes of the membrane.

### 3.2. 2D Mode Maps

**Figure 3:**
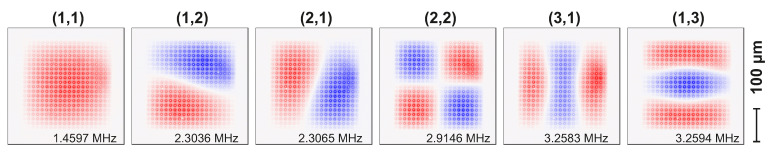
Normalized mode maps (real part) of the first six modes of the square SiN membrane acquired in a single, simultaneous, measurement with our PLL method. The release holes are visible as an array of dots across the entire surface of the structure. Starting frequencies are indicated in each map; further properties are listed in Table A1.

Using our method, the first six modes were measured simultaneously while scanning the membrane in the x and y direction, resulting in the 2D mode maps shown in Figure 3. The first mode at 1.46 MHz is indeed the (1,1) mode, and the second one at 2.30 MHz is the (1,2) mode. Although some modes are slightly distorted compared to Equation (1) (See Appendix B for discussion), one can still easily recognize them. In addition, note that fine details, such as the release holes (1 μm radius) are clearly visible in these high-resolution maps.

**Figure 4:**
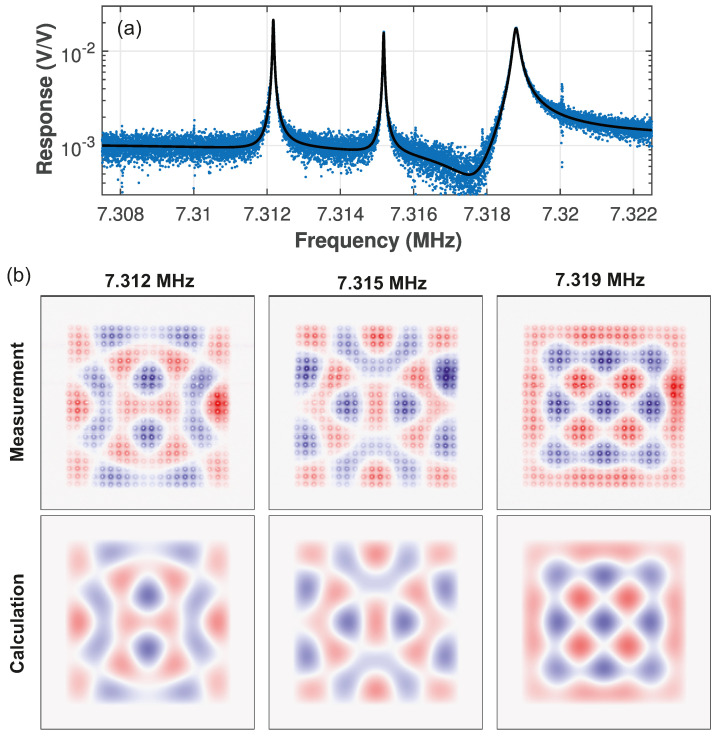
(**a**) Driven response near $f_{5,5}=f_{1,7}=f_{7,1}$. The triplet is fitted using three standard harmonic oscillator responses taking crosstalk into account (Appendix A) [32] (black line). (**b**) Measured (left) and calculated (right) mode maps. The weights from the linear fitting are $\left\{w_{5,5},w_{1,7},w_{7,1}\right\}\propto \left\{0.561,-0.506,0.656\right\}$, {0.817,0.260,−0.515}, and {0.105,0.694,0.712} for the left, middle, and right peak, respectively. The fit uncertainty in the weights is 0.002.

When modes are degenerate, a superposition of them is also an eigenmode and, a priori, it is not clear what their modes would look like. Mode mapping is thus crucial to understand the nature of the resonance. Equation (2) shows that the (5,5), (1,7), and (7,1) mode are triple degenerate and that these are expected around 7.3 MHz. Figure 4a shows three distinct peaks near this frequency. Their mode maps in Figure 4b indicate that, unlike the modes in Figure 3, their shapes are not directly given by Equation (1); instead, they show a much richer spatial structure. With the resolution of our mapping, it is possible to quantitatively determine the contributions of each individual mode to the superpositions. For this, the mode shape is written as $u\left(x,y\right)=w_{5,5}\xi_{5,5}\left(x,y\right)+w_{1,7}\xi_{1,7}\left(x,y\right)+w_{7,1}\xi_{7,1}\left(x,y\right)$ and the weights $w_{m,n}$ are determined by linear fitting to the experimental mode shapes. The results in the bottom row of Figure 4b show good agreement with the experiment, including the structure of nodal lines (white) and the variation in amplitude at the different anti nodes. Finally, note that the modes have very different damping rates γ, as seen from the peak width in Figure 4a (see also Table A1). It is known that the clamping losses depend on the displacement field near the edge [8,22,30,31]. Looking at the first two mode shapes shows alternating positive (red) and negative (blue) displacements near the edge of the membrane, whereas the third one (cf. the one with increased damping) has the same sign everywhere along the edge (red only); the radiation of acoustical energy into the supports would be very different. This explanation for their different linewidths would be difficult to obtain without our high-resolution mode maps with phase information.

## 4. Conclusions

In conclusion, we have presented a fast method to map the amplitude and phase of vibrational modes under realistic conditions. Our method is based on an improved PLL and is robust against frequency drift, phase jumps, and nodal lines and outperformed traditional methods. The novelty and advantage lies in the combination of high resolution, sensitivity, tracking using a PLL, and its robustness against sign changes of the mode shape when crossing nodal lines. We have illustrated the technique using a high-stress Si_3_N_4_ membrane, where degenerate modes were unambiguously identified. Up to six modes can be mapped simultaneously and from high-resolution mode maps the individual weights of superposition modes could be determined, and insights in the clamping loss mechanisms were obtained.

## Figures and Tables

**Figure A2:**
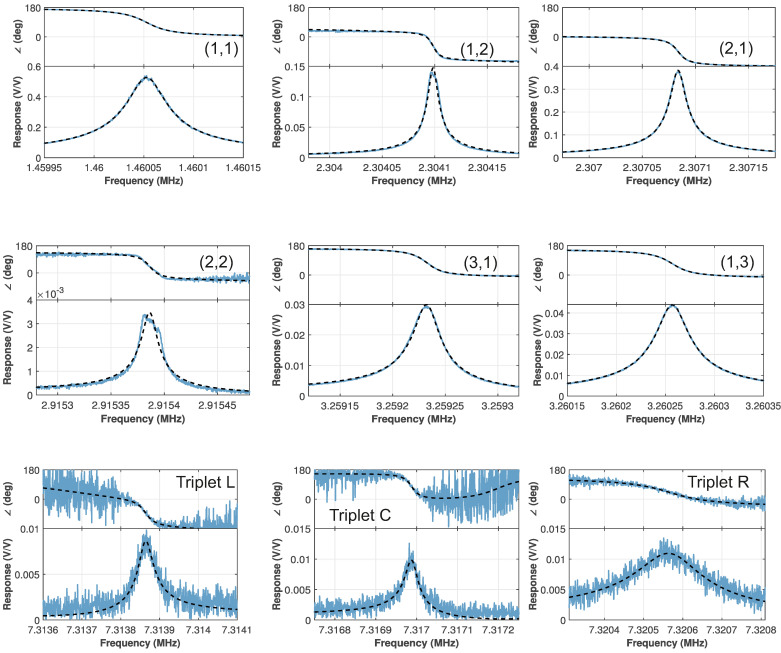
Driven responses of all the modes used in this work. For the first 6 modes, the span is 200 Hz, whereas for the triplet the span is 500 Hz. The gray curves are measured using the LIA, whereas the red curves are the fits of Equation (A1) to the data. The measurement settings and fit results are indicated in Table A1.

**Figure A3:**
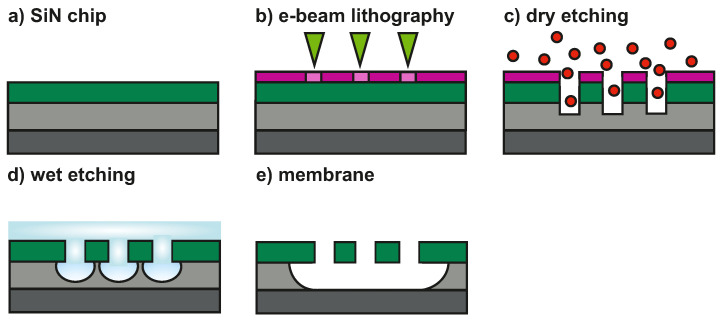
Schematic illustration of the nanofabrication process flow of the sample. (**a**) The sample is diced from a wafer with 330 nm Si_3_N_4_ on 3300 nm SiO_2_ on a Si substrate. (**b**) The holes of the membrane are patterned by electron beam lithography. (**c**) The holes are dry etched by reactive ion etching. (**d**) The membrane is released by wet etching with buffered hydrofluoric acid (light blue). (**e**) Under the released membrane a cavity is formed, resulting in the interference utilized for measuring the mode shapes.

## Appendix A. Demodulation, Response, and Mode Shape

In this appendix the details on the actuation and detection, lock-in operation and demodulation, response function, and their relation to the mode maps are discussed.

### Appendix A.1. Excitation and Detection

As illustrated in Figure 1b, the LIA outputs a voltage at (angular) frequency ω with amplitude $V_{\mathrm{out}}$: $V_{\mathrm{output}}\left(t\right)=V_{\mathrm{out}}\cos\left(\omega t\right)$. This is applied to the piezo (“plate c252 x40 y9 t0,1 eCuNi” from Physik Instrumente GmbH & Co. KG with a thickness of 0.1 mm, resulting in a fundamental thickness-mode resonance at 21.3 MHz. The membrane modes discussed in this work are thus all within the excitation bandwidth of the piezo), resulting in an oscillating inertial force on the membrane, which induces vibrations. These result in a modulation of the reflected power that is detected using the photodetector and converted to a voltage by the low-noise amplifier. This voltage goes to the input of the LIA and contains the same frequency as the output: $V_{\mathrm{input}}\left(t\right)=V_{\mathrm{in}}\cos\left(\omega t+\phi \right)$. Here, $V_{\mathrm{in}}$ is the amplitude at the excitation frequency ω and ϕ is the phase difference between output and input signals.

### Appendix A.2. Demodulation

Demodulation with the LIA can be seen as a calculation of the quadratures $V_{X}$ and $V_{Y}$ using $V_{Z}\left(t\right)=\langle 2V_{\mathrm{input}}\left(t\right)\exp\left(-i\omega t\right)\rangle \equiv V_{X}+iV_{Y}$. Here, the angled brackets indicate low pass filtering using the demodulation bandwidth. The complex demodulated voltage $V_{Z}\left(t\right)$ can also be expressed in its magnitude and phase $V_{Z}\left(t\right)=\left|V_{Z}\left(t\right)\right|\exp\left(i∠V_{Z}\left(t\right)\right)$, where *∠* denotes the argument of a complex number. For the aforementioned signal $V_{\mathrm{input}}\left(t\right)=V_{\mathrm{in}}\cos\left(\omega t+\phi \right)$, one obtains $V_{X}=V_{\mathrm{in}}\cos\left(\phi \right)$ and $V_{Y}=V_{\mathrm{in}}\sin\left(\phi \right)$ so that $|V_{Z}\left(t\right)|$ is $V_{\mathrm{in}}$ and $∠V_{Z}\left(t\right)=\phi$. Due to the low-pass filtering, the demodulated signals (“quadratures”) are only slowly (compared to ω) varying in time; their sampled values are indicated in the main text with the index *n*.

### Appendix A.3. Response and Mode Shape

In the linear response regime used in our experiments, the measured signal $V_{\mathrm{input}}\left(t\right)$ is proportional to the drive amplitude $V_{\mathrm{out}}$. Hence, the responses in the main text are normalized by $V_{\mathrm{out}}$ and their units are, thus, V/V. In particular, we define $Z\equiv X+iY\equiv V_{Z}/V_{\mathrm{out}}$. This overall system response can be written as a product of the frequency responses of the individual components: $Z\left(\omega \right)=G\left(\omega \right)R\left(\omega \right)\;\partial P/\partial U_{m,n}\;H_{m,n}\left(\omega \right)A\left(\omega \right)$. Here, *A*, *R*, and *G* are the responses of the piezo transduction (“actuation”), photodetector (“responsivity”), and amplifier (“transimpedance gain”), respectively. Although on larger scales, such as in the overview in Figure 1c, signatures of the frequency-dependent piezo response can be seen [20], in a narrow span around the resonance frequency of interest, $\omega_{0}$, all these responses are approximately constant and the ω dependence is omitted. In contrast, $H_{m,n}\left(\omega \right)$ is the harmonic oscillator response function that transduces the piezo vibrations via the inertial force into the vibrational amplitude $U_{m,n}$ of the (m,n) mode, which varies strongly near the eigenfrequency $\omega_{0}=\omega_{m,n}$ (In principle, the response would be a sum over all modes $H_{\mathrm{total}}\left(\omega \right)=\sum_{m,n}H_{m,n}\left(\omega \right)$ but for simplicity it is assumed here that only a single mode (m,n) gets excited. In Figure 4a and Figure A1 the combined responses of, respectively, three and two modes are fitted to the data). Finally, $\partial P/\partial U_{m,n}$ is the change of the reflected laser power with displacement of the entire mode $U_{m,n}$ [24]. Importantly, this quantity depends on the position of the laser spot: it is largest at anti-nodes and zero at a node. Using Equation (1) and applying the chain rule, it can be rewritten as $\partial P/\partial U_{m,n}=\partial P/\partial u\times \xi_{m,n}\left(x,y\right)$. Now ∂P/∂u is independent of the position as it quantifies the change in reflected power *P* for a given displacement *uat the readout position*(x,y) (Further refinements in the readout model could include the finite spot size of the laser by writing $\partial P/\partial U_{m,n}$ as a two dimensional convolution of the beam shape and the mode shape). Combining all this, gives $Z\left(x,y,\omega \right)=Ce^{i\alpha }H_{m,n}\left(\omega \right)\xi_{m,n}\left(x,y\right)$ for some proportionality constant *C* and complex angle α. This shows that the mapped normalized demodulated signal at constant frequency Z(x,y|ω) is directly proportional to the mode shape $\xi_{m,n}\left(x,y\right)$. Measuring *Z* as function of the readout position (x,y) using any of the methods in the main text thus enables mapping the mode shape $\xi_{m,n}$.

### Appendix A.4. Harmonic Oscillator Response

The harmonic oscillator response function H(ω) has the largest magnitude at the resonance frequency $\omega =\omega_{0}$ [24]. There, its phase is $∠H(\omega_{0})=-\pi /2$ so that on resonance, the total phase of $Z(\omega_{0})$ becomes α−π/2modπ where the last term comes from the sign of $C\xi_{m,n}\left(x,y\right)$. This phase is typically used as the setpoint for the PLL, $\phi_{\mathrm{sp}}$, as it locks ω to $\omega_{0}$, thereby maximizing the magnitude of the signal. For this reason, the value of α is needed for every mode, which is determined by fitting the driven frequency response Z(ω|x,y) measured at a fixed position. In particular, they were fitted using the expression:

(A1) $$Z\left(\omega \right)=z_{\max}e^{i\alpha }\frac{\omega_{0}\gamma }{\omega_{0}^{2}-\omega^{2}+i\omega \gamma }+z_{X}e^{i\phi_{X}},$$

where $z_{\max}$ is the peak value and γ the damping rate which is related to the quality factor $Q=\omega_{0}/\gamma$. The $z_{X}$ term includes a small amount of electrical crosstalk [20,32] (cf. the gray line in Figure 1c), which result in Fano-like resonances.

Both during the demodulation (as a setting in the LIA), as well as in postprocessing, an additional “analyzer” phase ψ can be set. In the former case, this corresponds to exp(−iωt)→exp(−iωt−ψ) for the aforementioned calculation of the quadratures. We use the latter, though, which performs the transformation

(A2) $$\left(\begin{array}{c} X^{\prime }\\ Y^{\prime } \end{array}\right)=\left(\begin{array}{cc} \cos\psi & \sin\psi \\ -\sin\psi & \cos\psi \end{array}\right)\left(\begin{array}{c} X\\ Y \end{array}\right).$$

By setting the analyzer phase equal to the setpoint of the phase-lock loop, $\psi =\phi_{\mathrm{sp}}$, the $X^{\prime }$ quadrature (cf. the real part of $Z^{\prime }$) contains the mode shape, whereas $Y^{\prime }$ (cf. the imaginary part of $Z^{\prime }$) is related to the error $e_{j}$.

So far, the analysis has been for a single frequency ω. The Zurich Instruments HF2 lock-in amplifier with multi-frequency kit option can, however, generate an excitation signal with six different frequencies and subsequently demodulate the input signal at all of these. This enables measurements on six modes *simultaneously*, as employed in, for example, Figure 3.

## Appendix B. Mode Properties

In this section, additional data on the different modes of the membrane are given.

**Table A1:** Overview of the excitation power, setpoint, and fit parameters (see Figure A2) for the modes used in the main text. The fit uncertainty in the resonance frequency is below 3 Hz for all modes, but the exact value drifts over the course of time.

Mode	Excitation	Frequency	Q	γ/2π	$z_{\max}$	α	$\phi_{\mathrm{sp}}$
	(dBm)	(MHz)	($10^{3}$)	(Hz)	(V/V)	(rad)	(°)
(1,1)	−45	1.460053	39.0±0.1	37.42±0.05	0.527	3.111±0.001	87
(1,2)	−50	2.304098	250.0±2.4	9.22±0.09	0.149	0.594±0.007	−68
(2,1)	−50	2.307084	167.4±0.4	13.78±0.04	0.384	0.012±0.002	−98
(2,2)	−35	2.915386	208.3±3.9	14.00±0.26	$3.5\cdot 10^{-3}$	2.283±0.014	−104
(3,1)	−35	3.259232	140.2±0.6	23.24±0.09	0.030	2.856±0.003	−111
(1,3)	−40	3.260257	104.7±0.2	31.14±0.05	0.044	2.788±0.001	−20
Triplet L	−35	7.313864	199.8±9.6	36.61±1.76	$9.1\cdot 10^{-3}$	0.172±0.037	−68.8
Triplet C	−35	7.316988	238.0±12.2	30.74±1.58	$9.7\cdot 10^{-3}$	2.842±0.039	−263.3
Triplet R	−35	7.320566	45.0±1.3	162.81±4.66	0.011	2.262±0.029	49.8

A zoom of the response near the (3,1) and (1,3) modes is shown in Figure A1. Figure A2 shows zooms of the modes discussed in the main text and Table A1 lists their properties. It is noted that due to temperature variations in the lab, the values for the exact resonance frequencies may differ slightly between measurements. This drift, in combination with the relatively long duration of response function measurements, resulted in a clear distortion of the (2,2) mode. A similar measurement with the NWA showed a regular response and yielded γ/2π=5.22±0.02, which corresponds to Q=559 k.

Figure 3 showed the measured two dimensional mode maps of the first six modes of the square membrane. Some of the maps do not show completely straight nodal lines as expected from the theory. This may be an effect of the finite bending rigidity of the membrane [33] and is confirmed using finite-element simulations. Another observation is that $f_{1,2}<f_{2,1}$ whereas $f_{1,3}>f_{3,1}$. This means that the breaking of the degeneracy between the (m,n) and (n,m) modes for m≠n is not caused by different side lengths of the membrane (cf. a rectangular instead of a square shape) and that more subtle effects play a role here.

## Appendix C. Sample Fabrication

Figure A3 explains the sample fabrication steps. First, a 6×10mm chip is diced from a 4″ wafer with 330nm LPCVD Si_3_N_4_ on 3300 nm SiO_2_ on a Si substrate (a) and is cleaned. Electron-beam resist (ZEP 520A) is applied and the holes of the membrane are patterned by electron beam lithography using a Nanobeam nB5 (b). Next, the holes are dry etched into the SiO_2_ layer using reactive ion etching (c) with a SF_6_/CHF_3_ inductively-coupled plasma (ICP). The membrane is released (d) by wet etching with buffered hydrofluoric (BHF) acid for 130 min. This ensures that the circular cavities around the holes are fully overlapping and that the Si layer is fully exposed. Note that BHF has good selectivity to Si [34], resulting in a smooth surface underneath the membrane. Then, the chip is transferred into water and subsequently into isopropanol and finally dried using a critical point dryer. As shown in Figure A3e, the SiN membrane (green) is now freehanging above the silicon substrate (dark gray). Between the released membrane and the substrate a low-finesse optical cavity is formed whose resonance wavelength depends on the separation between these two layers, giving rise to the displacement-dependent reflection utilized for measuring the mode shapes. The fabrication process has high yield and is very repeatable; different samples have very similar resonance frequencies and quality factors for nominally identical devices.
